# Endometriosis in Pregnancy: A Case Report

**DOI:** 10.7759/cureus.28749

**Published:** 2022-09-03

**Authors:** Lynzee Janowitz, Rachel Cooper-Mercado, Maria Soto

**Affiliations:** 1 Obstetrics and Gynecology, Lake Erie College of Osteopathic Medicine, Bradenton, USA; 2 Obstetrics and Gynecology, AdventHealth Sebring, Sebring, USA

**Keywords:** pregnancy, obstetrics&gynaecology, emergency obstetrics, fallopian tube mass, pregnancy-related pelvic pain, endometrioma, preterm labor, endometriosis

## Abstract

Women with endometriosis often present with pelvic pain and are at an increased risk of preterm labor. In this report, we discuss the case of a 27-year-old G2P1 at 29 weeks of gestation who presented to the ED with severe abdominal pain after being seen several times since 24 weeks of gestation in the obstetrics emergency triage with complaints of abdominal pain. Labs showed anemia with an elevated white blood cell (WBC) count and elevated liver function tests (LFTs) and CA-125. Due to intense pain, imaging was unavailable at the time of presentation, but the patient had a new mass the prior week, which was 9 cm in diameter and appeared to be in the uterine cavity. The patient was in preterm labor with advanced cervical dilation with the baby in double footling breech presentation, and hence a C-section was performed demonstrating a left hemorrhagic tubal mass determined to be an endometrioma on pathology. The patient had an uncomplicated postoperative course and was discharged on her third hospital day. Our case report focuses on a unique presentation and the pathophysiology of endometriosis. Endometriosis can present in various ways, leading to a delay in diagnosis and treatment. Endometriosis can create a significant burden on a woman’s health and financial status, and hence it is important to continue to study its complex presentation, in search of more effective, affordable, and non-invasive treatments.

## Introduction

Endometriosis is a common condition in which endometrial tissue implants outside of the uterus. It affects 2-10% of American women between the ages of 25 and 40 years [1]. It most commonly involves the ovaries, fallopian tubes, and peritoneal tissue. Among the various manifestations of endometriosis are pelvic pain, dyspareunia, and heavy/irregular periods. Infertility is believed to occur in up to 30-50% of women with endometriosis [2]. Although its pathogenesis is not clear, endometriosis is classically believed to be caused by retrograde menstruation in which menstrual blood travels through the fallopian tubes into the peritoneal cavity during menstruation. In recent years, other possible theories of the underlying pathophysiology of endometriosis have been proposed. Another current theory involves coelomic metaplasia, which hypothesizes that endometriosis is derived from hormonal stimulation of cells of the mesothelial lining of the peritoneum, causing these cells to transform into endometrium-like cells [3]. Additionally, another theory proposes the idea that estrogen causes the proliferation of ectopic endometrial lesions, while there is also an apparent progesterone resistance preventing the normal shedding of the endometrial lining. Other theories include those of oxidative stress and inflammation, autoimmune disease, apoptosis suppression, and genetic predisposition to endometriosis [3].

Complications of endometriosis during pregnancy are postulated to be caused by several different processes that are currently being studied, as endometriosis in pregnancy is rare. One such process is the impaired reorganization and decidualization of the uterine spiral vessels [4]. Since decidualization of the uterine spiral arteries depends on the effect of progesterone, the lack of this effect can lead to the involution of the vessels and bleeding in pregnant patients with endometriosis. This disruption of the physiology of pregnancy can lead to the complications seen in pregnant patients with endometriosis, such as miscarriage, ectopic pregnancy, hemorrhage during pregnancy, premature rupture of membranes, preterm birth, infants small for gestational age, and cesarean delivery [5]. Additionally, endometriosis is a state of chronic inflammation known to cause adhesions due to increased fibrin deposition compared to fibrin breakdown. Adhesions can create tension in surrounding structures as the uterus expands during pregnancy. This continuous inflammatory process causes an increase in inflammatory mediators such as prostaglandins, which leads to cervical ripening and collagen degradation, as well as tissue dysfunction, which could explain the higher risk of preterm labor and of vessel rupture, respectively [4].

## Case presentation

**Figure 1:**
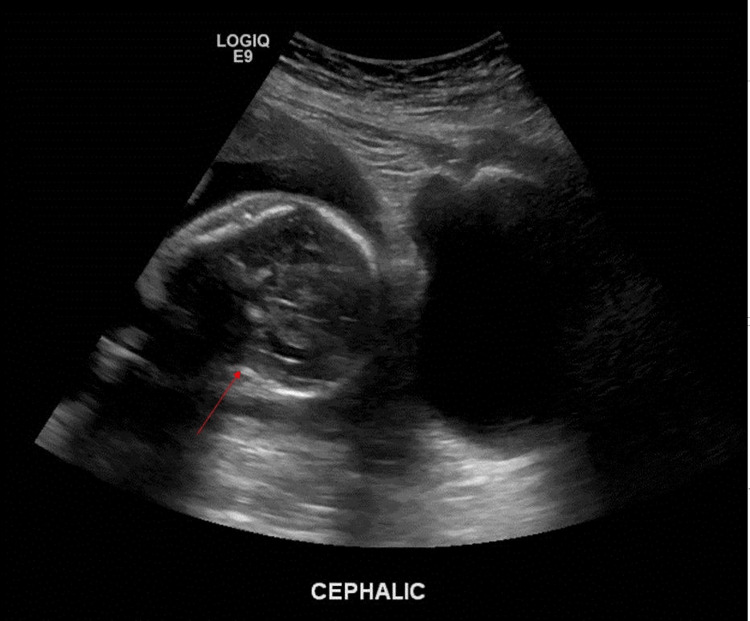
Ultrasound at 24 weeks of gestation shows a single viable uterine pregnancy (red arrow)

A 27-year-old G2P1 woman presented to the ED at 24 weeks of gestation with severe abdominal pain. The patient had a past medical history of vaginal bleeding during her first pregnancy in 2016, cholelithiasis, and iron deficiency anemia. Otherwise, her medical history was unremarkable without any preterm births or labor complications. Due to the severity of her abdominal pain, she was admitted to the hospital for an extensive workup including labs, which were unremarkable, and an MRI abdomen, which was positive for cholelithiasis in a nondistended gallbladder. Additionally, she received a cervical length and amniotic fluid index (AFI) check, resulting in an abdominal ultrasound that showed a single viable uterine pregnancy (Figure 1) with an AFI at the upper limit of normal and the cervix closed with a length of 3.5 cm.

**Figure 2:**
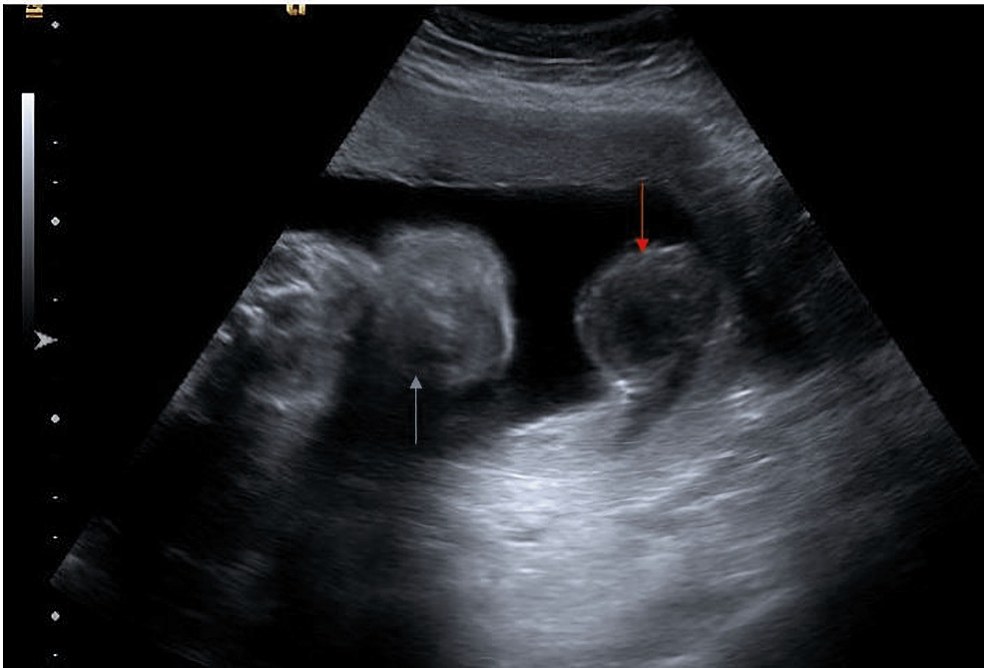
Ultrasound at 28 weeks The gray arrow shows the head of the fetus. The red arrow shows the left fallopian tube endometrioma

At 28 weeks of gestation, the patient presented to the ED with severe abdominal pain and heavy vaginal bleeding. Labs showed that the patient was anemic with a mildly elevated white blood cell (WBC) count and elevated liver function tests (LFTs) and CA-125. On pelvic ultrasound, an avascular 9-cm diameter mass was emanating from the posterior mid-left side wall of the uterus (Figure 2). She was admitted and administered Celestone 12 mg intramuscularly to ensure fetal lung maturity, magnesium sulfate 4 GM bolus followed by 2 GM per hour for fetal neuroprotection, and tocolysis to prepare for her transfer to a tertiary care center. The patient was discharged from the tertiary care center on her third hospital day.

**Figure 3:**
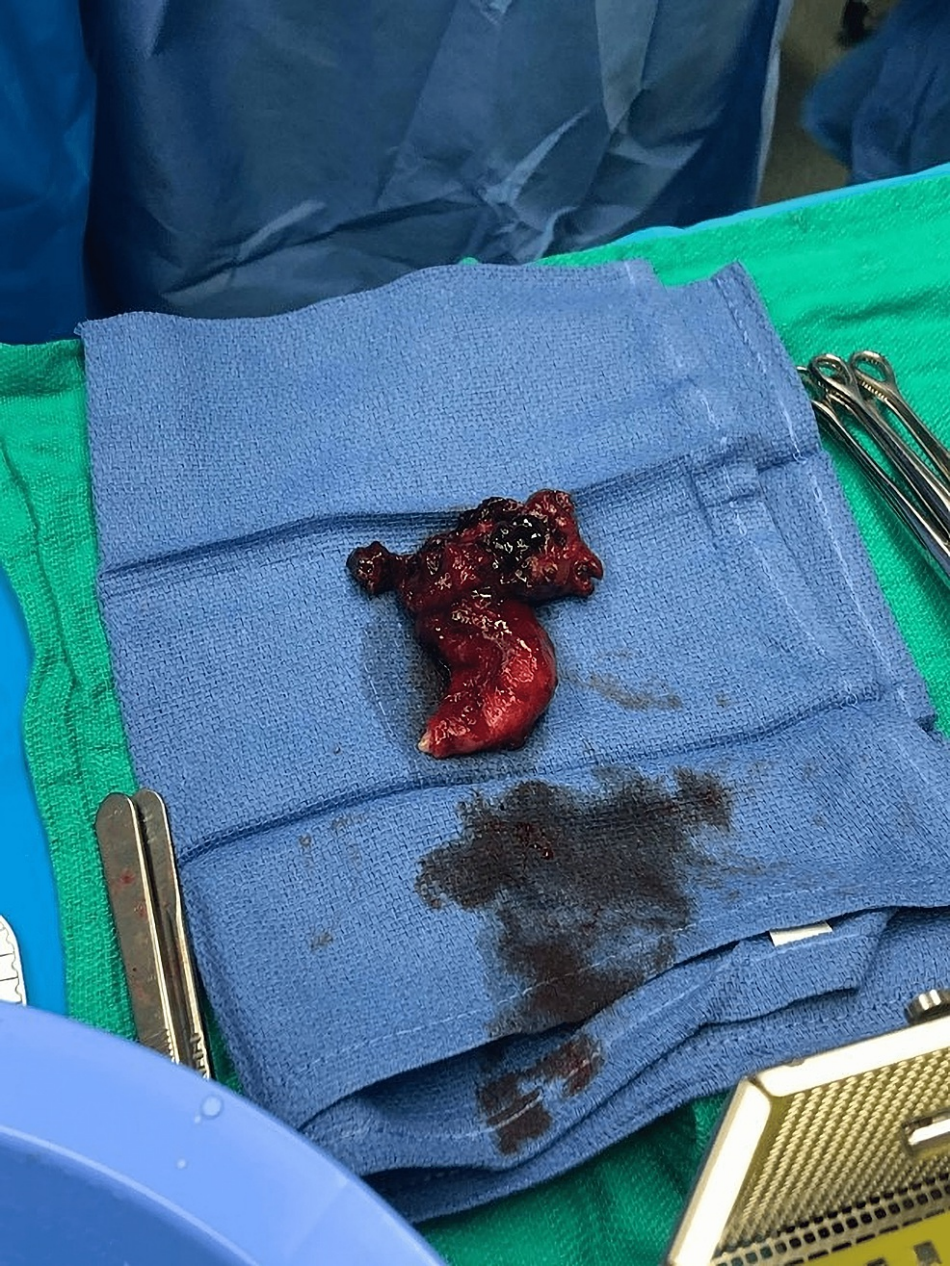
Left fallopian tube with endometrioma

The patient returned to the hospital one week later with severe abdominal pain. She was admitted to the hospital in preterm labor with advanced cervical dilation at 29 weeks of gestation. The baby was in double footling breech presentation, and hence the patient delivered via C-section in which a left tubal endometrioma (Figure 3) was resected along with the attached fallopian tube, leaving the remaining ovary intact. The pathology report showed the left fallopian tube with decidua, adhesions, hemorrhage, and mesothelial hyperplasia consistent with endometrioma with pregnancy-associated changes. The patient had an uncomplicated postoperative course and was discharged home on her third hospital day.

## Discussion

Even though one of the main causes of chronic pelvic pain is endometriosis, arriving at this diagnosis can be very time-consuming and costly. Symptoms can vary in presentation and severity depending on the location and spread, but many individuals can be asymptomatic and remain unaware of their condition until other problems arise, such as infertility. For some of the women who are able to conceive, pregnancy might offer a short reprieve from endometriosis because of the elevated levels of progesterone during pregnancy. Previous studies have shown that the use of progestin, a synthetic version of progesterone, can reduce endometriosis symptoms by inhibiting the proliferation of the endometrium. Unfortunately, symptoms often return postpartum, and may even worsen due to high levels of estrogen.

Our patient did not have a prior diagnosis of endometriosis. Due to her symptoms of abdominal pain at 24 weeks of gestation, an MRI of the abdomen was performed to rule out any bowel pathology; MRI was only remarkable for cholelithiasis in a nondistended gallbladder. Additionally, an ultrasound of the pelvis and duplex ultrasound of the abdomen were unremarkable, ruling out ovarian torsion. Our patient did not have a prior diagnosis of endometriosis, and hence her main complaints of pelvic and abdominal pain remained unexplained until a large pedunculated mass was seen on ultrasound four weeks later. The ultrasound report and the rapid growth of the mass suggested a possible uterine neoplasm, but the mass was later found to be an endometrioma. This finding of an enlarging endometrioma is rare, as one study showed that only 20% of patients with ovarian endometrioma had an enlargement of the mass during pregnancy [5]. Currently, there is scarce data explaining growth patterns of endometriosis during pregnancy as most studies at this point show a regression or cessation of growth of ovarian endometriomas during pregnancy, as explained above.

In non-emergent situations, endometriosis can initially be treated with watchful waiting, non-steroidal anti-inflammatory drugs (NSAIDs), and hormonal therapies such as estrogen-progestin oral contraceptive pills (OCPs) [6]. Hormonal OCPs are used to stop the process of ovulation, thereby decreasing menstruation and potentially alleviating the severity of symptoms. Gonadotropin-releasing hormone (GnRH) agonists are second-line medical agents used to decrease menstruation by acting on the hypothalamic-pituitary-gonadal axis to stop ovulation. The next step in treatment once conservative measures fail often involves laparoscopic surgery to remove endometrial implants, followed by hysterectomy if laparoscopy fails to relieve symptoms. Due to the emergent nature of our case, the only option for our patient was immediate delivery and removal of the hemorrhagic mass and tube. Although the patient and her child experienced a favorable outcome, this case highlights the need for further research on the clinical presentation and treatment of endometriosis.

## Conclusions

Studies have shown that endometriosis affects about 10% of women of reproductive age. It can cause chronic pelvic pain and infertility. It can present in a wide variety of ways in pregnant women, including miscarriage, ectopic pregnancy, and premature rupture of membranes. Our case had a unique presentation of an enlarging endometrioma leading to preterm labor and emergent C-section. The current medical treatments for endometriosis are mostly limited to NSAIDs, hormonal drugs, and surgery, which may have a limited impact on women with certain concurrent medical conditions, such as pregnancy, hypercoagulability, and certain cancers. Research is still ongoing to find novel ways to diagnose, monitor, and treat endometriosis.
